# Non-toxigenic *Vibrio cholerae* non-O1/non-O139 pseudo-bacteraemia in a neonate: A case report

**DOI:** 10.4102/sajid.v36i1.263

**Published:** 2021-07-22

**Authors:** Wentzel B. Dowling, Mené Van der Westhuyzen, Michele Haumann, Kessendri Reddy

**Affiliations:** 1 Division of Medical Microbiology and Immunology, Faculty of Health Sciences, Stellenbosch University, Cape Town, South Africa; 2 National Health Laboratory Service, Tygerberg Hospital, Cape Town, South Africa; 3 Department of Paediatrics, Paarl Hospital, Cape Town, South Africa

**Keywords:** *Vibrio cholerae*, pseudo-bacteraemia, non-toxigenic *Vibrio cholerae*, non-01/non-O139 *Vibrio cholerae*

## Abstract

Toxigenic *Vibrio cholerae* O1/O139 is causative of cholera, which is a well characterised potentially epidemic gastrointestinal disease. Less is known about the pathogenesis and clinical presentation of non-toxigenic *V. cholerae* non-O1/non-O139, although they are increasingly implicated in human disease globally, have been isolated from various South African water sources and can contaminate the environment. The authors describe a case of pseudo-bacteraemia with non-toxigenic *V. cholerae* non-O1/non-O139 in a neonate.

## Introduction and background

Toxigenic *Vibrio cholerae* (*V. cholerae*) is causative of cholera, a long-established gastrointestinal illness, which remains a potentially epidemic infectious disease with high mortality and morbidity.^1,2^ *Vibrio cholerae* has a widespread environmental aquatic reservoir and can cause human disease when water sources become contaminated with *V. cholerae*, especially in areas where there is poor sanitation infrastructure, unsafe drinking water, natural disasters or wars.^1,2,3^

The two main pathogenic *V. cholerae* serogroups, O1 and O139, have been described extensively in the literature, but less is known about the pathogenesis and clinical presentation of non-toxigenic *V. cholerae* non-O1/non-O139 infections.^4,5^

There are several virulence factors contributing to toxigenic *V. cholerae* pathogenicity: the two major virulence factors are cholera toxin and toxin co-regulated pilus (TCP).^1,2^ The cholera toxin gene, harboured on a temperate bacteriophage (CTXɸ), can be transmitted horizontally, although the timing of CTXɸ lysogeny, integration and replication is complex and poorly understood.^1,6,7^

Compared with the severe diarrhoeal disease caused mainly by the toxin-producing O1 and O139 serogroups, non-toxigenic *V. cholerae* non-O1/non-O139 have been reported to cause mild gastroenteritis, wound infections, ear infections, meningitis and bacteraemia.^4^ However, toxigenic *V. cholerae* non-O1/non-O139 such as *V. cholerae* serogroups O75 and O41, have also been described as a cause for diarrhoeal disease similar to cholera.^8^ Non-toxigenic *V. cholerae* bacteraemia has predominantly been described in adults with liver pathology (cirrhosis, alcoholism), haematological malignancies, diabetes mellitus and renal disease.^5^

The authors describe a case of pseudo-bacteraemia with non-toxigenic *V. cholerae* non-O1/non-O139 in a neonate at a secondary hospital in the Western Cape, South Africa (SA).

## Case

A 1-day old premature neonate was born by normal vertex delivery at 34 weeks’ gestation had a birthweight of 2040 g and Apgar scores of 8 and 9, respectively. Shortly after birth (day 1 of life) the patient developed mild respiratory distress secondary to grade 2 hyaline membrane disease, which was managed with the administration of surfactant and continuous positive air pressure (CPAP) ventilation. Infective markers (done on day 1 of life) included an increased C-reactive protein (CRP) of 53 µg/mL with a normal white cell count of 4.94 cells × 10^9^/L. A blood culture and lumbar puncture (LP) were performed on day 1 of life, to exclude sepsis in the context of respiratory distress. The patient was started empirically on ampicillin 150 mg 6 hourly and ceftazidime 90 mg 12 hourly intravenously on the same day because of a provincial shortage of cefotaxime. Following 1 day of CPAP, the patient remained afebrile and was stable on room air. On day 2 of life, the patient developed mild neonatal jaundice and borderline hypoglycaemia, which resolved after treatment with ultraviolet phototherapy and intravenous fluids.

The initial blood culture flagged positive after 48 hour incubation (on day 3 of life) and curved gram-negative bacilli were observed on the Gram stain and processed accordingly (see Microbiology investigation). No bacterial growth was detected, from a repeat blood culture (performed on day 5 of life), following 5 days of incubation. However, the patient was on empiric antibiotics at the time of blood culture collection. The cerebrospinal fluid (CSF) analysis was within normal value ranges. A follow-up CRP, taken on day 6 of life, was within normal limits (4 µg/mL). The patient’s antibiotic therapy was de-escalated to ampicillin 150 mg 6 hourly and gentamicin 15 mg once daily intravenously on day 3 of life as the patient was clinically stable. A 7-day antibiotic course was completed in total.

The patient received formula milk on day 1 and following successful latching, exclusive breastfeeding was instituted. The patient had no community exposure prior to discharge at day 8 of life. The mother reported no comorbidities, no recent exposure to shellfish, and no history of gastroenteritis. She reported washing the baby with tap water during the hospital stay (from day 1 of life) and consumed unboiled tap water at home. The exact timing of washing of the baby, in relation to the blood culture venepuncture (on day 1 of life), could not be elucidated. Further history of iatrogenic exposure to tap water or washing remains unclear. A stool sample taken from the mother (5 days after the baby’s birth) excluded the presence of *V. cholerae.*

As a result of the patient’s presentation, stable clinical course, absence of features suggestive of neonatal non-toxigenic *V. cholerae* non-O1/non-O139 infection (such as meningo-encephalitis) and *V. cholerae* not being endemic in the Western Cape, it was concluded that this was a non-toxigenic *V. cholerae* non-O1/non-O139 pseudo-bacteraemia.

## Microbiology investigation

**FIGURE 1:**
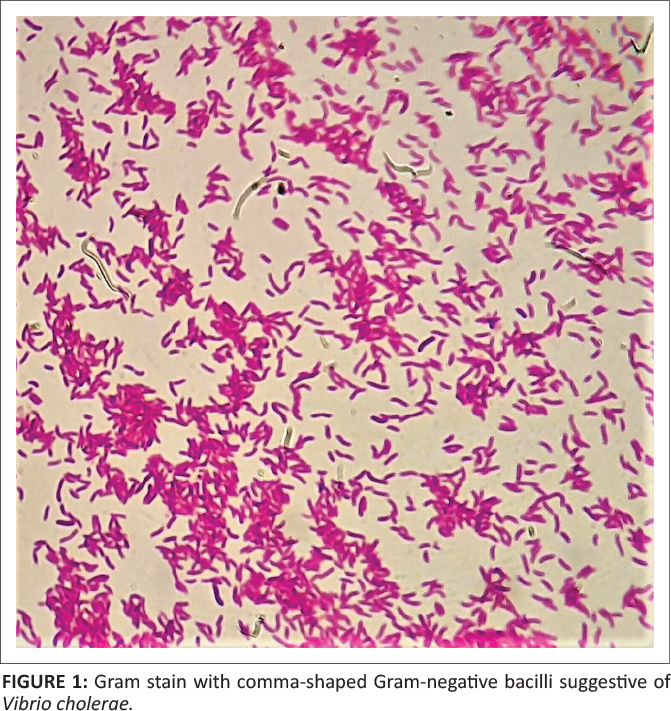
Gram stain with comma-shaped Gram-negative bacilli suggestive of *Vibrio cholerae.*

The blood culture bottle was incubated in the BacT/Alert 3D automated incubator (bioMérieux Inc., Marcy l’Etoile, France) and microbial growth was detected after 48 h with curved gram-negative bacilli observed on the Gram stain (see Figure 1). The blood culture broth was sub-cultured onto tryptose blood agar, cooked blood agar and MacConkey agar (without crystal violet) and incubated overnight in a 5% CO_2-_enriched atmosphere at 35 °C.

**FIGURE 2:**
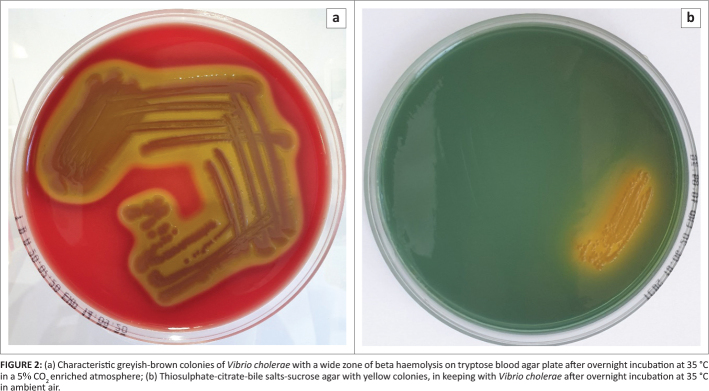
(a) Characteristic greyish-brown colonies of *Vibrio cholerae* with a wide zone of beta haemolysis on tryptose blood agar plate after overnight incubation at 35 °C in a 5% CO_2_ enriched atmosphere; (b) Thiosulphate-citrate-bile salts-sucrose agar with yellow colonies, in keeping with *Vibrio cholerae* after overnight incubation at 35 °C in ambient air.

Large dry greyish-brown colonies with beta-haemolysis grew on the tryptose blood agar plate (see Figure 2a), with corresponding growth on cooked blood agar and MacConkey agar. Automated biochemical analysis, using the VITEK 2 Gram-negative identification card (bioMérieux Inc., Marcy l’Etoile, France) identified *V. cholerae* with 99% confidence in identification.

A thiosulfate-citrate-bile salts-sucrose agar (TCBS) and bile aesculin agar were inoculated and incubated overnight (to assist in differentiating *V. cholerae* from Aeromonas species). The TCBS agar showed yellow colonies in keeping with *V. cholerae* (see Figure 2b), no aesculin hydrolysis was observed and oxidase test was positive. Baltimore Biological Laboratory (BBL) Crystal Enteric/Non-Fermenter identification system (Becton Dickinson Inc., USA) could not differentiate between *V. cholerae* and *Aeromonas hydrophila,* as these two bacteria are morphologically and biochemically very similar.

Proteomic analysis with the Vitek MS (bioMérieux Inc., Marcy l’Etoile, France) confirmed the identification of *V. cholerae.* The isolate was further tested at the National institute for Communicable Diseases (NICD) Centre for Enteric Diseases, with molecular methods that verified the isolate to be a non-toxigenic *V. cholerae* non-O1/non-O139 serogroup.

## Discussion

Toxigenic *V. cholerae* is endemic in many sub-Saharan African countries.^2^ In SA, sporadic imported cases are reported annually from neighbouring countries such as Zimbabwe.^2,9^ A few major cholera epidemics have been described in SA, with the largest outbreak being in 2008/2009 and only one SA study from the 1980s describing toxigenic *V. cholerae* bacteraemia in a neonate.^2,9,10^ Toxigenic *V. cholerae* O1/O139 is currently not endemic in the Western Cape with the last known cases coinciding with the 2008/2009 epidemic.^9^ Non-toxigenic *V. cholerae* non-O1/non-O139 is rare in neonates, with only eight previous published cases.^11,12,13,14^ The majority of these cases (7/8) presented with meningoencephalitis and had residual neurological deficits following infection.^11,12,13,14^) In the remaining case,^15^ the patient presented with fever, did not have a LP and demised in hospital.

This is in contrast with the clinical findings in our case, where the patient recovered well, had a normal CSF analysis, had a negative repeat blood culture (whilst on empiric antibiotics) and had a repeat CRP that was not elevated.

Our case represents a non-toxigenic *V. cholerae* non-O1/non-O139 pseudo-bacteraemia and highlights the likely presence of non-toxigenic *V. cholerae* non-O1/non-O139 in water sources from healthcare environments in the Western Cape. It also highlights the complexity of the laboratory diagnosis of non-toxigenic *V. cholerae* non-O1/non-O139, as *Vibrio* species can be incorrectly identified as the more commonly isolated *Aeromonas* species because of biochemical similarities.^7,16^ Important laboratory tests to differentiate between *V. cholerae* and *Aeromonas* species are the observation of comma-shaped Gram-negative bacilli on Gram stain, the presence of yellow colonies on TCBS agar, a positive string test, a lack of aesculin hydrolysis and ‘shooting star’ motility on wet preparation or darkfield microscopy.^16,17^

It is important to note that only serogroups O1 and O139 have been implicated in pandemics^7^ and that these serogroups are distinct from the diverse spectrum of non-toxigenic *V. cholerae* non-O1/non-O139 found in marine and estuarine environments.^2^ Environmental studies in SA have reported both toxigenic (unknown serogroups) and non-toxigenic *V. cholerae* non-O1/non-O139 isolates from various water sources.^18,19,20^ Transformation from non-O1/non-O139 serogroups to O1/O139 serogroups and toxigenic transformation, has been reported, but is rare in the environment.^6^

In our case study, the source of the *V. cholerae* isolate, causing pseudo-bacteraemia, is poorly elucidated but is likely from an environmental or aquatic reservoir. The most likely source of contamination is thought to be the hospital water supply system. Contaminated water could have been introduced onto the skin either by washing or a breakdown in aseptic technique whilst performing the blood culture.

As both toxigenic and non-toxigenic *V. cholerae* strains have an ubiquitous aquatic niche, the maintenance and monitoring of water and sewerage systems and the prompt notification of clinical cholera disease^1,3^ is crucial to prevent the resurgence of cholera in SA. It is also important to remain vigilant to potential cholera outbreaks, even when non-toxigenic strains are isolated from clinical specimens.

Our case of non-toxigenic *V. cholerae* pseudo-bacteraemia aims to increase awareness of the role of non-toxigenic *V. cholerae* strains in the clinical setting. As cholera is infrequent in SA, it is imperative to ensure continued education on the presentation and laboratory diagnosis of cholera, the diverse clinical presentations (and potential contaminant role) of non-toxigenic *V. cholerae* and the implications of a *V. cholerae* diagnosis on patient treatment and public health response, for both toxigenic and non-toxigenic *V. cholerae* cases.
